# Cu(OH)_2_ and CuO Nanorod Synthesis on Piezoresistive Cantilevers for the Selective Detection of Nitrogen Dioxide

**DOI:** 10.3390/s18041108

**Published:** 2018-04-05

**Authors:** Laurent Schlur, Manuel Hofer, Ahmad Ahmad, Karine Bonnot, Mathias Holz, Denis Spitzer

**Affiliations:** 1 Nanomatériaux pour les Systèmes Sous Sollicitations Extrêmes (NS3E), UMR 3208 ISL/CNRS/UNISTRA, French-German Research Institute of Saint-Louis, 5, rue du Général Cassagnou, 68300 Saint-Louis, France; karine.bonnot@hotmail.fr (K.B.); denis.spitzer@isl.eu (D.S.); 2 Nano analytik GmbH, Ehrenbergstraße 1, 98693 Ilmenau, Germany; hofa1@gmx.at (M.H.); ahmad.ahmad@stud.tu-ilmenau.de (A.A.); m.holz@nanoanalytik.net (M.H.)

**Keywords:** piezoresistive sensors, nanostructured sensors, CuO/Cu(OH)_2_ nanorods, NO_2_ detection, selective detection, explosives detection

## Abstract

Self-controlled active oscillating microcantilevers with a piezoresistive readout are very promising sensitive sensors, despite their small surface. In order to increase this surface and consequently their sensitivity, we nanostructured them with copper hydroxide (Cu(OH)_2_) or with copper oxide (CuO) nanorods. The Cu(OH)_2_ rods were grown, on a homogeneous copper layer previously evaporated on the top of the cantilever. The CuO nanorods were further obtained by the annealing of the copper hydroxide nanostructures. Then, these copper based nanorods were used to detect several molecules vapors. The results showed no chemical affinity (no formation of a chemical bond) between the CuO cantilevers and the tested molecules. The cantilever with Cu(OH)_2_ nanorods is selective to nitrogen dioxide (NO_2_) in presence of humidity. Indeed, among all the tested analytes, copper hydroxide has only an affinity with NO_2_. Despite the absence of affinity, the cantilevers could even so condensate explosives (1,3,5-trinitro-1,3,5-triazinane (RDX) and pentaerythritol tetranitrate (PETN) on their surface when the cantilever temperature was lower than the explosives source, allowing their detection. We proved that in condensation conditions, the cantilever surface material has no importance and that the nanostructuration is useless because a raw silicon cantilever detects as well as the nanostructured ones.

## 1. Introduction

Microcantilevers used for the development of highly sensitive sensor probes have been massively studied in the literature for the last 20 years. Such cantilevers are identical to the atomic force microscopy cantilevers and are sensitive to temperature [1] and humidity [2] variations. They are also able to detect several molecules (explosives [3,4], Volatile Organic Compounds (VOCs) [5,6], chemical warfare agents [7,8]). A modification of the bending and of the resonance frequency of the cantilever during the adsorption of the molecules on its surface allows the detection [9]. This technology presents sensitivities down to parts per billion (ppb) and even below parts per trillion (ppt) thresholds with high accuracy and reliability, allowing both the quantification and the identification of an analyte in a mixture of compounds by functionalizing the cantilever surface for selective detection [7,10].

For the moment, the sensitivity of these micromechanical devices is limited by their small surface area. To overcome this problem, the cantilever surface can be nanostructured with TiO_2_ nanotubes [10], ZnO nanorods or nanotubes [11,12], carbon nanotubes [13,14], mesoporous silica [15] or the cantilever surface is anodized in order to have a porous surface [16]. Such nanostructured cantilevers are able to detect really low concentration of explosives (ppt scale) [10,15] and several VOCs [15,16].

Copper hydroxide (Cu(OH)_2_) and cupric oxide (CuO) nanostructures are of great interest due to their numerous application potentials. Few attempts were made to use the base-centered monoclinic CuO [17,18,19,20] and the base-centered orthorhombic Cu(OH)_2_ [21,22] nanostructures to detect VOCs, H_2_S, explosives and glucose. Several syntheses were developed to synthesize CuO one-dimensional (1D) nanowire/nanotubes arrays: thermal treatment [23], hydrothermal synthesis [24] and wet chemical synthesis [25]. Copper hydroxide 1D structures can be obtained electrochemically [26] and by wet chemistry [27,28].

The approach developed here offers highly sensitive microcantilevers with enhanced surface area due to the 1D nanostructures. We use the cantilevers with a piezoresistive readout developed by Rangelow et al. [29,30,31]. Such readout does not need any alignment and adjustment of a Laser comparing with the optical readout cantilevers. For the first time the surface of these piezoresistive cantilevers is nanostructured with Cu(OH)_2_ and CuO nanorods. The synthesis procedure developed and herein described allows the mass production of nanostructured piezoresistive cantilevers. After an optimization of the detection tests parameters, the sensitivity of these cantilevers toward explosives and VOCs were tested.

## 2. Materials and Methods

### 2.1. Growth of CuO Nanorods on the Piezoresistive Cantilevers

**Figure 1:**
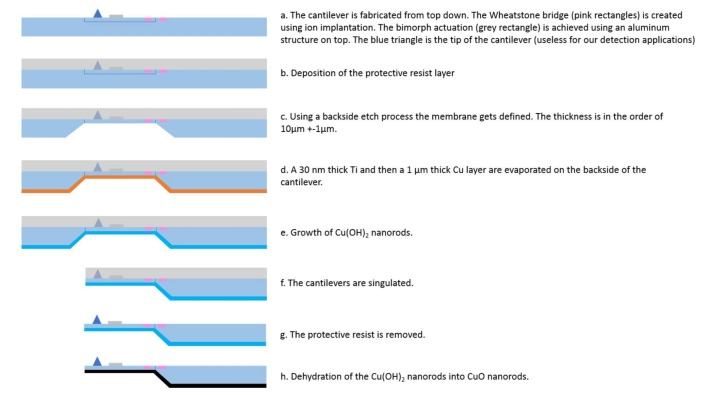
Fabrication process of the nanostructured piezoresistive cantilevers.

The method of cantilever nanostructuring used in this work describes the incorporation of the nanostructure synthesis to the fabrication process of the cantilevers on the wafer level and not to the single cantilever. This method allows the mass production of nanostructured piezoresistive cantilevers. The synthesis procedure was performed on copper thin layer deposited on a 4″ wafer before etching and connecting of the cantilever. The different fabrication steps of the nanostructured cantilevers are visible on Figure 1.

The prestructured cantilevers [32,33,34,35] were first made on the front side of the wafer (Figure 1a). As the chemical solution used for the nanostructures growth is highly basic, a 10 µm protective resist layer was deposited to the previous processed cantilever structures in order to prevent the destroying dissolution of the piezoresistive Wheatstone bridge including its electrical metal based connectors and the bi-material electro-thermal actuator used to actuate the cantilever (Figure 1b) [34,36,37]. The Wheatstone bridge enables the measurement of the resonance frequency and the actuator allows the oscillation of the cantilever. Before the copper layer was evaporated on the wafer, a membrane was etched until a final thickness of only 10 ± 1 µm (Figure 1c). Therefore, the back side of the wafer had a 1000 nm thick thermally grown SiO_2_ layer. After lithography, it was structured using a combination of an anisotropic plasma Reactive Ion Etching process with a gas mixture of 50 sccm Ar, 50 sccm CHF_3_ at a chamber pressure of 30 mTorr, 150 W power, and a back side He cooling at 10 °C [38]. The membrane was achieved with a Tetramethylammonium hydroxide (TMAH) etching process. The 20% concentrated TMAH bath was heated up to 90 °C, resulting in an etch rate of 180 nm per minute.

After the preparation of the membrane a 30 nm thin titanium layer, a 1 µm thick copper layer was evaporated under low vacuum pressure on the back side of the wafer (Figure 1d). The presence of titanium improves the copper adhesion on the wafer. The next step was the growth of the copper hydroxide (Cu(OH)_2_) nanorods (Figure 1e). This synthesis comprised of two successive reactions, described in detail by L. Schlur et al. [28]. The copper coated wafer was horizontally placed upside-down (the copper face orientated to the bottom) in a crystallizing dish containing an aqueous solution of sodium hydroxide (8.31 × 10^−2^ mol/L) and ammonium persulfate (4.15 × 10^−3^ mol/L). The distance between the wafer and the bottom of the container was fixed to 0.5 mm. After 15 min of reaction at 20 °C, the wafer was transferred to a second crystallizing (for the second reaction) dish containing the same reactants, where it was horizontally placed upside-down with a distance of 5 mm to the bottom. In this second beaker the sodium hydroxide and the ammonium persulfate concentrations were higher, than in the first one. They were equal to 2.66 mol/L and 1.33 × 10^−1^ mol/L respectively. The growth time and temperature of the second reaction were the same as for the first one. Subsequently, the wafer was rinsed with distilled water and dried under N_2_ gas flow.

A semiautomatic method was used to separate the single sensors out of the wafer and to mount them on a special holder for the final wire bonding (Figure 1f). The protective resist was removed with commercially available Allresist Remover AR 300-76 (Figure 1g).

Finally, the copper (II) hydroxide nanostructures present on the surface of the cantilever were dehydrated into copper oxide (Figure 1g). For this purpose, the nanostructured cantilever was placed in the middle of a furnace. Subsequently the sample was annealed with a heating rate of 1 °C/min to 200 °C, under static air. The cantilever was kept at 200 °C for 1 h before natural cooling down to room temperature.

The morphology and the size of the nanostructures were studied by Scanning Electron Microscopy (SEM), using a FEI Nova NanoSEM 450 equipped with a Field Emission Gun. The resistance tests of the fully integrated Wheatstone bridge created by ion implantation and the resonance frequency measurements of the cantilever were done with a test bench at Nanoanalytik GmbH.

### 2.2. Explosives and Volatile Organic Compounds Detection

The detection measurements were performed on two explosives: 1,3,5-trinitro-1,3,5-triazinane (RDX) and pentaerythritol tetranitrate (PETN). The explosive source consisted of a tube filled with 70 glass beads covered by the desired explosive, and closed with deactivated glass wool. The tube has a length of 2.5 cm and an external diameter of 6 mm. The tube and the glass wool, which were purchased from Restek^®^ are treated to avoid the adsorption of molecules. To cover all the surface of the 70 glass beads with RDX, 20 mg of RDX were mixed with the beads in a vortex mixer for two seconds. For PETN, 10 mg of explosive were used and the vortex lasted 15 s.

Detection tests were also done on several volatile compounds: toluene, benzene, trichloroethylene, tetrachloroethylene, xylene, acetaldehyde, acrolein, and nitrogen dioxide. Each of these compounds were contained in individual permeation tubes produced by Owlstone Inc. (Norwalk, CT, USA). A permeation tube is composed with porous polymer closed at both ends and filed with the desired analyte. This tube releases a constant concentration of the analyte under a given temperature.

**Figure 2:**
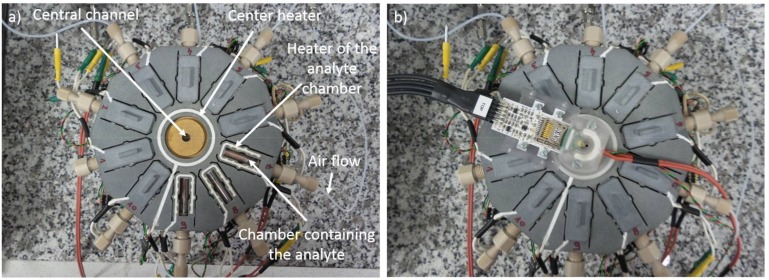
Vapor generator device (**a**) without; (**b**) with the cantilever chamber.

These different sources were placed in a homemade vapor generator apparatus as shown in Figure 2.

The vapor generator is composed of 10 individual chambers, nine of which can contain each an analyte (explosive, or a volatile organic compound or a pollutant, etc.). The last one used as a reference, remains empty. With its 10 chambers, this vapor generator was designed to generate mixtures (tested molecules with different pollutants traditionally present in air), though in this paper no mixture was tested. An air supply is connected to each of these 10 chambers. The other end of the chambers was connected to a central channel on the top of which the cantilever chamber containing the piezoresitive cantilever was placed. The cantilever chamber, the central channel and all 10 chambers can be individually heated. The cantilever is continually exposed to a 50 mL/min airflow. Two types of air were used: either a “dry air” having a water concentration lower than 500 ppb or a humid air which flows through a water bubbler before entering inside the detection chamber. In order to detect possible leaks problems, the airflow was constantly controlled by a flowmeter fixed at the outlet of the device (on the cantilever chamber lid (not visible on Figure 2). Until the stabilization of the cantilever resonance frequency, 50 mL/min of dry or humid air was injected inside the cantilever chamber via the reference chamber and the central channel (Figure 2). After this stabilization phase, the airflow of the reference chamber was switched off. In the same time, the airflow of the chamber containing the desired explosive or VOC is switched on. From that moment the cantilever is in contact with the desired explosive or VOC vapors. A decrease of the resonance frequency means that the cantilever detects the tested molecule. After 3, 5, or 10 min (depending on the tested molecule), the air flow is switched again from the analyte chamber to the reference chamber. For one detection experiment, the same air “type” (dry or humid) was always used in the reference chamber and in the chamber containing the tested analyte.

The temperature of the analyte chamber determines the concentration of the analyte vapors. Different temperatures were used in order to test different concentrations. The concentration was determined theoretically and confirmed by chromatography. For all the measurements, the reference chamber and the chamber containing the analyte source were at the same temperature. The temperature of the cantilever was also varied in order to measure in condensation or non-condensation conditions. When the cantilever has a lower temperature than the analyte source, the analyte can condensate on the cantilever surface if all the thermodynamic conditions are met. In the opposite case, the cantilever is only able to detect the analyte if there is an affinity between the copper oxide nanostructures and the tested molecules.

## 3. Results and Discussion

### 3.1. Piezoresistive Cantilevers Covered with CuO Nanorods

**Figure 3:**
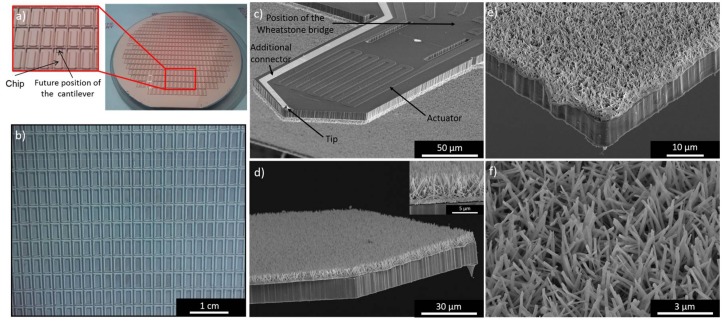
Photography of the 4″ wafer used for the production of piezoresistive cantilevers: (**a**) before the nanostructures growth; and (**b**) after the Cu(OH)_2_ nanorods synthesis; (**c**–**f**) Scanning electron microscopy (SEM) tilted view of the nanostructured cantilevers; (**c**) Cantilever face showing the Wheatstone bridge and the actuator; (**d**–**f**) Cantilever face which is nanostructured.

The previously described synthesis allows the production of 450 piezoresistive cantilevers with one single batch. Before the nanostructures grew, the back side of the wafer surface was partially etched and covered by the 1 µm thick copper layer (Figure 3a). This surface was divided into 450 rectangles. One rectangle was composed of the chip of the cantilever and the cantilever itself (which was not visible as it was in the bulk of the wafer), so each rectangle corresponded to one future sensor. Figure 3b shows a part of the same wafer after the nanostructures synthesis. The blue coloration visible on the surface, indicates the presence of Cu(OH)_2_. After nanostructure growth, the wafer was etched again in order to obtain 450 individual cantilevers, with Cu(OH)_2_ nanorods on their back side and with the actuator, and the Wheatstone bridge on the front side (Figure 3c–f). The cantilevers had a mean length, width and thickness of 350 µm, 150 µm and 11 µm respectively. In order to dehydrate copper hydroxide nanostructures into copper oxide each cantilever was then annealed at 200 °C.

One face of the cantilever consisted of the bimorph actuator and the Wheatstone bridge. The implemented resistors of the Wheatstone bridge were not visible (Figure 3c) as they were incorporated in the cantilever, but the four connectors of the bridge were visible. On this face of the cantilever, the tip and an additional connector which were not used in this paper were also visible.

The other face of the cantilever (Figure 3d–f) was homogeneously covered with nanorods having a length of 4.59 ± 0.33 µm and a diameter of 163 ± 39 nm. The nanorods density was approximately 7.2 × 10^8^ cm^−2^, corresponding to ca. 400,000 nanorods per microcantilevers. The presence of the CuO nanorods on the piezoresistive cantilever surface increased the surface area by a factor of 17 compared with the raw cantilevers. The length, diameter and density of the nanorods were the same on all the cantilevers issued from the same wafer. It is possible to produce several hundreds of identical nanostructured piezoresistive cantilevers with only one chemical synthesis. The 1D oxides nanostructures synthesized on the surface of cantilevers by other teams [10,11,12] are generally comparable to those synthesized in this article: the same diameter, a sorter length (around 1 µm), and a higher density (10 times higher in some cases). In this article, the lower density was compensated by a higher length of the nanostructures. In order to obtain Cu(OH)_2_/CuO nanorods with the same characteristics as in the literature, the concentration of sodium hydroxide and ammonium persulfate in the first reaction (see experimental section) had to be decreased, which resulted in a decrease in the nanostructure length and an increases in the density (as proved by L. Schur at al. [28]).

In our previous work [28], the same chemical conditions (reaction time and reactants concentration) as those used here allowed the growth of Cu(OH)_2_ nanotubes and not of nanorods. In this previous work, containing all the nanostructures characterization (XRD, SEM, TEM), the authors proved that the first 15 min of chemical reaction allows the growth of Cu(OH)_2_ nanorods. The role of the second reaction which also lasts 15 min dissolves the center of the nanorods into nanotubes. Here, only nanorods were present on the wafer surface and no nanotubes were present because the surface of the wafer was 81 times bigger in this paper than in the previous one. The increase of the wafer surface increases the dissolution time of the nanorods center into nanotubes. In order to obtain nanotubes the second reaction time should be increased.

The cantilevers efficiencies were verified by measuring the resistance values of the Wheatstone bridge and the Al-bimorph heater structure. For all the tested cantilevers produced from the same wafer, the resistance of a single resistor was within a spectrum of 2.5 kΩ ± 100 Ω, whereas the resonance frequency varied between 105 to 130 kHz. 

### 3.2. Detection Results

**Figure 4:**
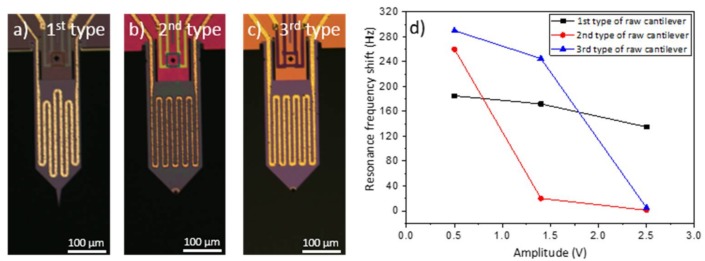
Optical microscopy pictures of the (**a**) first; (**b**) second and (**c**) third type of cantilever used to determine; (**d**) the variation of the resonance frequency shift with the cantilever vibration amplitude. The black, red and blue curves visible on (**d**) correspond to the resonance frequency shift of the first, second and third type of cantilever when 217 ppb of pentaerythritol tetranitrate (PETN) were generated during 3 min in condensation conditions. The vibration amplitude was measured at the maximum of the resonance peak.

Before using the nanostructured piezoresistive cantilevers, different tests were made with raw cantilevers in order to optimize the detection conditions. Three different type of raw cantilevers having different surfaces (first type: 38,000 µm^2^, second type: 47,000 µm^2^, third type: 46,000 µm^2^) and resonance frequencies (first type: 23 kHz, second type: 60 kHz, third type: 80 kHz) were used (Figure 4a–c). The cantilevers were each exposed for 3 min to a vapor of 217 ppb of PETN. The detection performances were measured for each in terms of the resonance frequency shift resulting from the detection of PETN molecules captured on the surface of the cantilever. The influence of the cantilever vibration amplitude on the detection performances was studied (Figure 4d). The vibration amplitude was measured at the maximum of the resonance peak. The measurements were performed in condensation condition i.e., for a temperature of the PETN source (85 °C) higher than the cantilever temperature (65 °C).

A resonance frequency shift due to the detection of 217 ppb of PETN in condensation condition was visible for the three raw cantilevers, except for the second and the third cantilever when the amplitude was equal to 2.5 V. For all the three type of raw cantilevers, the resonance frequency shift decreased, when the amplitude increased. For a given resonance frequency, the angular speed of the cantilever increased when the cantilever oscillation amplitude increased. The increase of the oscillation amplitude and of the angular speed prevented the deposition of the PETN molecules on the cantilever surface. For the following detection tests, the amplitude of the cantilever was fixed to 0.5 V in order to have the highest resonance frequency shift, and so to be as sensitive as possible. For lower values the signal became too noisy. As the air flow was perpendicular to the cantilevers surface, one possible solution to avoid the effect of the oscillation amplitude, and so to increase the cantilever sensitivity, was to use cantilevers able to vibrate in their in-plane flexural mode instead of cantilevers vibrating in the conventional out-of-plane flexural mode [39]. The authors did not try to confirm this hypothesis, because the cantilevers used were fabricated to vibrate out-of-plane.

Figure 4d also shows that for a given amplitude value, the resonance frequency shift of the three raw cantilevers was different and that the evolution of the three decreasing curves was also not the same. These differences cannot be easily explained; several parameters are probably involved. The main potential parameters are: the surface area of the cantilever (first type: 38,000 µm^2^, second type: 47,000 µm^2^, third type: 46,000 µm^2^), the resonance frequency (first type: 23 kHz, second type: 60 kHz, third type: 80 kHz). None of these three types of raw cantilevers were used in the following detection results because for a given amplitude value, the obtained detection results depend totally on the type of cantilever (Figure 4d). Raw silicon cantilevers similar to the nanostructured ones were then used. The Cu(OH)_2_ nanostructures present on the surface of nanostructured cantilevers were dissolved in a nitric acid solution (1 × 10^−1^ mol/L) during 45 min in order to have raw silicon cantilevers comparable to the nanostructured ones. After the nanostructure dissolution, the resonance frequency was approximately 15 kHz higher than before, because of the cantilever mass loss.

**Figure 5:**
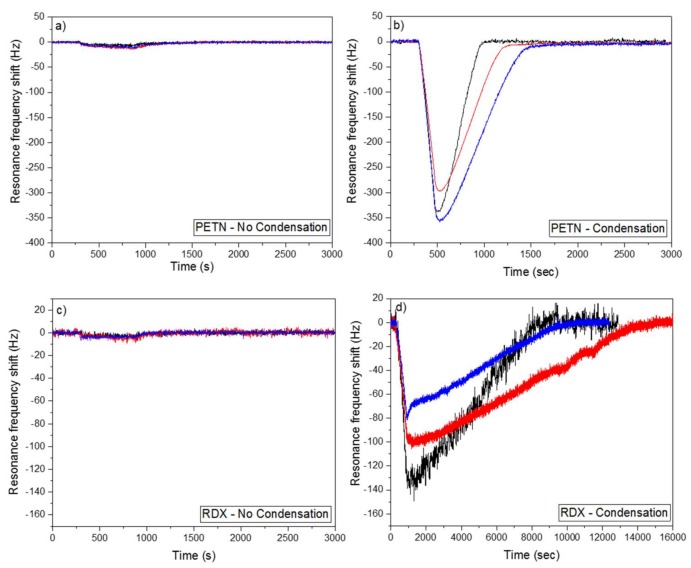
Resonance frequency shift of a raw (black), a Cu(OH)_2_ nanostructured (blue) and a CuO nanostructured (red) cantilever exposed during (**a**) 10 min or (**b**) 3 min to 217 ppb of PETN or (**c**); (**d**) during 10 min to 21 ppb of 1,3,5-trinitro-1,3,5-triazinane (RDX) . The measurements were performed (**a**); (**c**) in non-condensation condition or (**b**); (**d**) in condensation conditions. The cantilevers were exposed to the explosives five min after the beginning of the experiments. The air flow contained a water concentration lower or equal to 500 ppb.

The raw, the Cu(OH)_2_ nanostructured, and the CuO nanostructured cantilevers were exposed for 10 min to 217 ppb of PETN or to 21 ppb of RDX carried by a “dry air” flow. The measurements were performed in non-condensation conditions with a temperature of the explosives sources and of the cantilevers equal to 85 °C and 90 °C respectively (Figure 5a,c). In condensation conditions (Figure 5b,d) the temperature of the cantilevers was fixed at 65 °C and the cantilevers were exposed for 3 min to 217 ppb of PETN, or for 10 min to 21 ppb or RDX, with a vibration amplitude of the cantilever equal to 0.5 V.

In non-condensation conditions for all cantilever types (raw, Cu(OH)_2_ nanostructured and CuO nanostructured), very small shifts of the resonance frequency were observed during the injection of PETN or RDX. The shifts were too small to be considered as a detection signal, and could be attributed by the authors to small differences in the airflow and temperature values in the reference chamber and in the chamber containing the explosives sources. The absence of considerable resonance frequency shifts in non-condensation conditions means that neither PETN nor RDX are detected by all types of the cantilever (raw, Cu(OH)_2_ nanostructured and CuO nanostructured). The same results are obtained with an air flow saturated with water (figure not shown). An absence or a poor affinity between the tested explosives and copper hydroxide or copper oxide could explain these results. The absence of visible detection does not mean that the tested sensors are not able to detect higher concentrations of PETN or RDX.

In condensation conditions, the condensation of explosives on the cantilever surface can explain the resonance frequency shifts that are observed for the three type of cantilevers (raw, Cu(OH)_2_ nanostructured and CuO nanostructured). For PETN, the shifts were equal to 330 Hz for the raw cantilever, 338 Hz for the Cu(OH)_2_ nanostructured cantilever and 280 Hz for the CuO nanostructured cantilever. For RDX, the raw, the Cu(OH)_2_ and CuO cantilevers had a resonance frequency shift of 130 Hz, 80 Hz and 95 Hz respectively. For one type of explosive, the shift values of the three cantilevers were relatively close. Moreover, for the same experiment, which was repeated several times, a variation of the resonance frequency shift equal to 30% was observed (not shown here). Taking into account this information, the three tested cantilevers (raw, Cu(OH)_2_ and CuO) were equally sensitive to RDX or PETN. In this case, the nanostructure of cantilever with copper-based nanorods does not improve the explosives detection performance in condensation conditions contrary to the results obtained by Spitzer et al. [10], in non-condensation conditions. In Spitzer et al.’s work, the nanostructures improve the detection performances compared to non-nanostructured cantilevers, due to an affinity between the nanostructures and the detected molecules, which is not the case here. In condensation conditions, if there is no affinity between the cantilever and the analyte, the material of the cantilever surface also does not seem to be important, as the same detection results are obtained with a raw silicon cantilever, a Cu(OH)_2_ cantilever, and a CuO cantilever. In condensation conditions, the detection results depend on the apparent surface of the cantilever (i.e., the length and the width) and not on the surface roughness. Compared to the raw cantilever, the resonance frequency of the nanostructured cantilevers takes more time to return to the baseline after the interruption of the explosive injection. Indeed, in the case of PETN, the resonance frequency of the raw and the nanostructured cantilever returns to its initial value at a rate of 54 Hz/min and 29 Hz/min respectively. For RDX, the desorption rate is also more than two times slower for the nanostructured cantilevers (0.5 Hz/min) than for the raw one (1.2 Hz/min). The nanostructures act like traps that slow down the evaporation.

**Figure 6:**
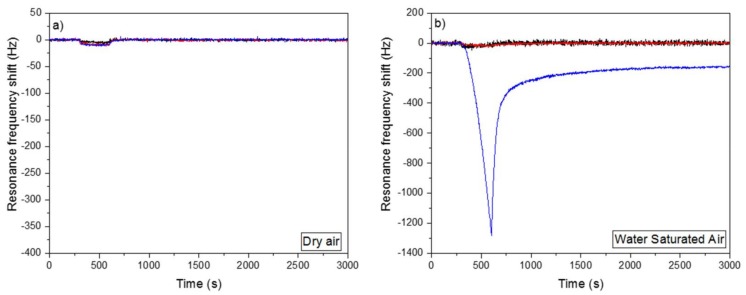
Response of a raw (black), a Cu(OH)_2_ nanostructured (blue) and a CuO nanostructured (red) cantilever to 45 ppm of NO_2_ in non-condensation conditions (Source temperature 61 °C, Cantilevers temperature 80 °C). (**a**) All the experiment (stabilization, detection and post detection phases) was done with a “dry air” flow containing 500 ppb of water; (**b**) All experiments were done with air flow saturated with water. After five minutes of stabilization, the cantilevers were in contact with NO_2_ over 5 min.

The affinity between the cantilevers and nitrogen dioxide has also been tested (Figure 6). The three types of cantilevers were in contact with 45 ppm of NO_2_ over 5 min. The NO_2_ vapors were carried either with a “dry air” flow having a water concentration lower than 500 ppb (Figure 6a) or with an air flow which passed through a water bubbler (Figure 6b). The cantilever temperature (80 °C) was higher than the NO_2_ source (61 °C) so no condensation was possible.

The raw and CuO nanostructured cantilevers have no affinity with nitrogen dioxide whatever the water concentration in the air flow. The small shift of the resonance frequency observed during the injection of NO_2_ was due to a small variation in the air flow and in the temperature.

In absence of water the Cu(OH)_2_ nanostructured cantilever also did not detect NO_2_, but in presence of water vapors the same cantilever was able to detect NO_2_. For five minutes, the resonance frequency shift was close to 1300 Hz. Contrary to numerous gas sensors, the presence of water is here an advantage and not a drawback. Water plays an important role in the detection of NO_2_. However, the presence of NO_2_ is also essential, as during the first five minutes of the experiment (stabilization phase) only water is present in the air flow and the Cu(OH)_2_ nanorods detect nothing. C. England et al. [40], showed that the simultaneous presence of nitrogen dioxide and water vapors allows the formation of nitric acid. Nitric acid reacts easily on the cantilever surface with copper hydroxide in order to form copper nitrate (Cu(NO_3_)_2_) and water. Both reactions are described in Equations (1) and (2). 

4NO_2(g)_ + 2H_2_O_(g)_ +O_2(g)_ ↔ 4HNO_3(g)_ (1)

Cu(OH)_2(s)_ + 2HNO_3(g)_ ↔ Cu(NO_3_)_2(s)_ + 2H_2_O_(l)_ (2)

After the exposure to NO_2_, the resonance frequency increases again because water molecules formed on the cantilever surface during Reaction 2 evaporate. Despite this increase, the resonance frequency does not return to its initial value because on the nanostructures surface, copper hydroxide is replaced by copper nitrate, which is heavier. As the sensing material (copper hydroxide) is consumed, the sensitivity of the sensor is affected. After five cycles of adsorption/desorption, the resonance frequency shift is equal to 300 Hz (instead of 1300 Hz for the first cycle) (figure not shown). However, the sensor can be regenerated in a basic solution containing sodium hydroxide.

**Figure 7:**
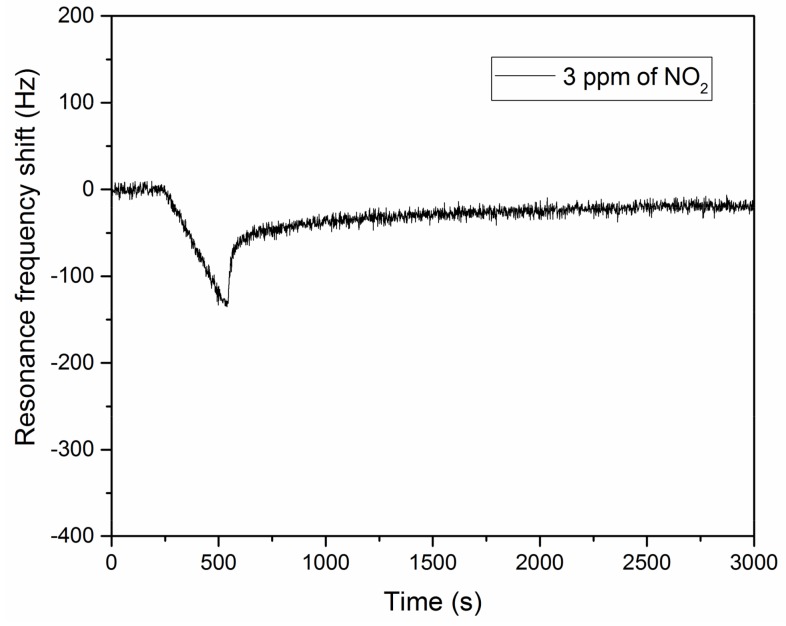
Resonance frequency shift of a Cu(OH)_2_ nanostructured piezoresistive cantilever exposed to 3 ppm of NO_2_ in non-condensation conditions (cantilever 55 °C, NO_2_ source 23 °C). All the experiment (stabilization, detection and post detection phases) were done with an air flow saturated with water. After five minutes of stabilization, the cantilever was in contact with NO_2_ over 5 min.

The Cu(OH)_2_ nanorods in the presence of water can detect easily 45 ppm of NO_2_ (Figure 6b). The same cantilever is also able to detect 3 ppm of NO_2_ (in non-condensation conditions), which was the lowest concentration we could generate with our device and with this permeation tube (Figure 7). The NO_2_ vapors were carried with an air flow which passed through a water bubbler. The resonance frequency shift after five minutes of exposure was equal to 135 Hz.

**Figure 8:**
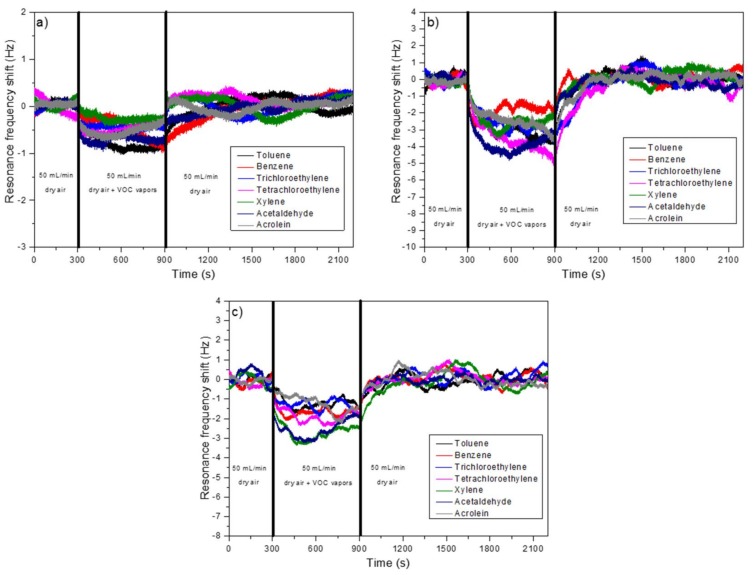
Resonance frequency shift of a (**a**) raw; (**b**) Cu(OH)_2_ nanostructured and (**c**) CuO nanostructured cantilever exposed during 10 min to 28 ppm of toluene (black), 20 ppm of benzene (red), 155 ppm of trichloroethylene (blue), 39 ppm of tetrachloroethylene (pink), 20 ppm of xylene-p (green), 553 ppm of acetaldehyde (dark blue) and 2 ppm of acrolein (grey). The measurements were performed under condensation conditions. The cantilevers were exposed to the VOCs five minutes after the beginning of the experiments and the air flow contained a concentration of water lower or equal to 500 ppb.

The Cu(OH)_2_ cantilever was exposed to several VOCs which are often present in the air in order to test its selectivity. The raw and the CuO nanostructured cantilevers were also exposed for 10 min to the same molecules (Figure 8). The measurements were performed in condensation conditions. The cantilever temperature is fixed to 50 °C and the VOCs source are heated to 61 °C. At this temperature the cantilevers were exposed to 28 ppm of toluene, 20 ppm of benzene, 155 ppm of trichloroethylene, 39 ppm tetrachloroethylene, 20 ppm of xylene-p, 553 ppm of acetaldehyde, and 2 ppm of acrolein. 

Only small resonance frequency shifts were observed for all three cantilever types (raw, Cu(OH)_2_ or CuO) and all tested VOCs. The value of these shifts were not important enough to be considered as a detection signal. These very small shifts were attributed by the authors to small differences of the airflow and temperature values in the reference chamber, and in the chamber containing the VOCs sources. The presence of nanostructures increases the influence of the air flow on the cantilever stability, which is why the shift observed for nanostructured cantilevers was slightly more important. For tested concentrations, the VOCs were not detected by the raw or by the nanostructured cantilevers. VOCs do not condensate on the surface of the cantilevers (at the tested temperatures), and they have also no affinity with the materials present on the cantilevers surface. The nanostructures have also no utility for these tested VOCs.

The cantilever nanostructured with Cu(OH)_2_ was a selective sensor as for all the tested molecules it only detected NO_2_. So, in classical working conditions of a sensor (i.e., ambient temperature) the Cu(OH)_2_ detected only NO_2_ among all the tested molecules in this article, because in classical conditions no condensation (of explosives) was possible as the sources were also not heated.

## 4. Conclusions

The surface of piezoresitive cantilevers was nanostructured with copper hydroxide and copper oxide nanorods and tested for their affinity with respect to different VOCs and explosive vapors. The nanorod synthesis was not done directly on each individual cantilevers, but it was introduced in the cantilever fabrication process, which allows the nanostructuration of several hundreds of cantilevers with only one synthesis. The synthesis consists of the oxidation of a homogeneous copper layer, previously evaporated, in an alkaline aqueous solution containing Na(OH) and (NH_4_)_2_S_2_O_8_.

During the detection measurements, the cantilever amplitude value has to be as low as possible because oscillation amplitude values that are too high prevent the deposition of molecules on the cantilever surface. In the experimental conditions of this paper, the raw and CuO nanostructured cantilevers have no affinity with all the tested analytes. The Cu(OH)_2_ nanostructured cantilevers are selective sensors as they have only an affinity with NO_2_ and not with the other analytes (PETN, RDX, toluene, benzene, trichloroethylene, tetrachloroethylene, xylene-p, acetaldehyde, and acrolein). The presence of water vapor is indispensable in the detection of nitrogen dioxide by copper hydroxide. In condensation conditions, the two tested explosives (PETN and RDX) condensate on the surface of the raw, Cu(OH)_2_ and CuO nanostructured cantilevers. For one given explosive, the resonance frequency shift of the three types of cantilevers is the same, which means that in condensation conditions (if there is no affinity between the cantilever and the analyte) the cantilever surface material has no importance and nanostructuration is not required.

This work shows that highly affine nanostructured piezoresistive cantilever sensors can be designed and mass produced for detecting specific molecules. We have demonstrated that copper hydroxide nanostructures synthesized on piezoresistive cantilevers show a high affinity and selectivity to NO_2_ vapors. In non-condensation conditions, NO_2_ can also be selectively detected, even in the presence of other molecules containing NO_2_, such as nitro-based explosives which are not detectable in non-condensation conditions. This work constitutes a major step in understanding the physics and chemistry of detection using highly selective materials.
